# Supplementary material for: Circulating tumor DNA reveals complex biological features with clinical relevance in metastatic breast cancer
Source: Nat Commun. 2023 Mar 1;14:1157. doi: 10.1038/s41467-023-36801-9 (PMC9977734; doi:10.1038/s41467-023-36801-9)
Supplement: Supplementary file 1 — Supplementary Information [file 41467_2023_36801_MOESM1_ESM.pdf]

Supplementary Information File

Circulating Tumor DNA Reveals Complex Biological Features with Clinical Relevance in Metastatic Breast Cancer

Aleix Prat, Fara Brasó-Maristany, Olga Martínez-Sáez, Esther Sanfeliu, Youli Xia, Meritxell Bellet, Patricia Galván, Débora Martínez, Tomás Pascual, Mercedes Marín-Aguilera, Anna Rodríguez, Nuria Chic, Barbara Adamo, Laia Paré, Maria Vidal, Mireia Margelí, Ester Ballana, Marina Gómez-Rey, Mafalda Oliveira, Eudald Felip, Judit Matito, Rodrigo Sánchez-Bayona, Anna Suñol, Cristina Saura, Eva Ciruelos, Pablo Tolosa, Montserrat Muñoz, Blanca González-Farré, Patricia Villagrasa, Joel S. Parker and Charles M. Perou and Ana Vivancos

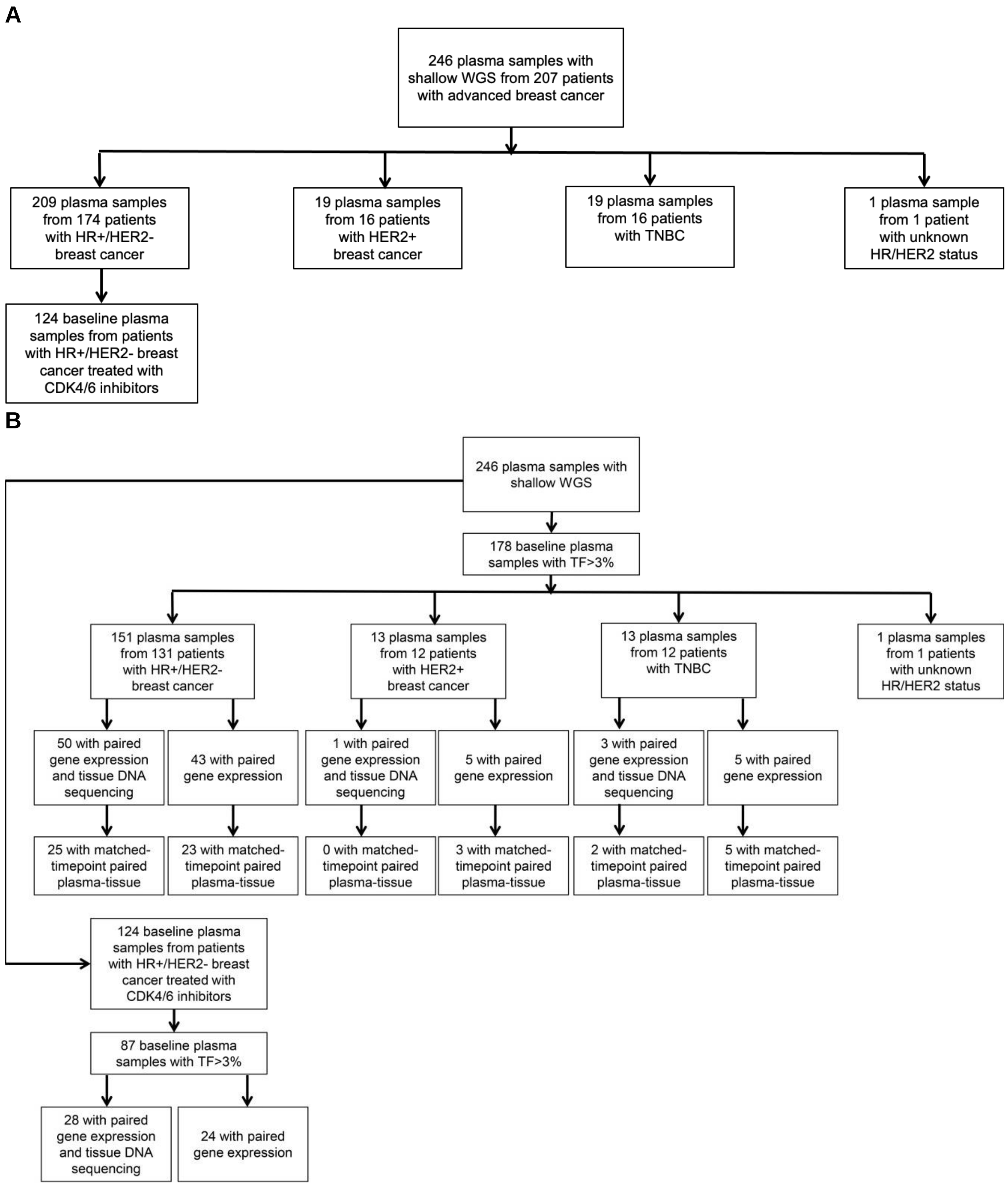

Supplementary Figure 1. Summary of samples used in the study. **(A)** shWGS was performed on 246 plasma samples from 207 patients with advanced HR+/HER2-negative breast cancer, HER2+ breast cancer, and TNBC. **(B)** Description of samples with TF>3%.

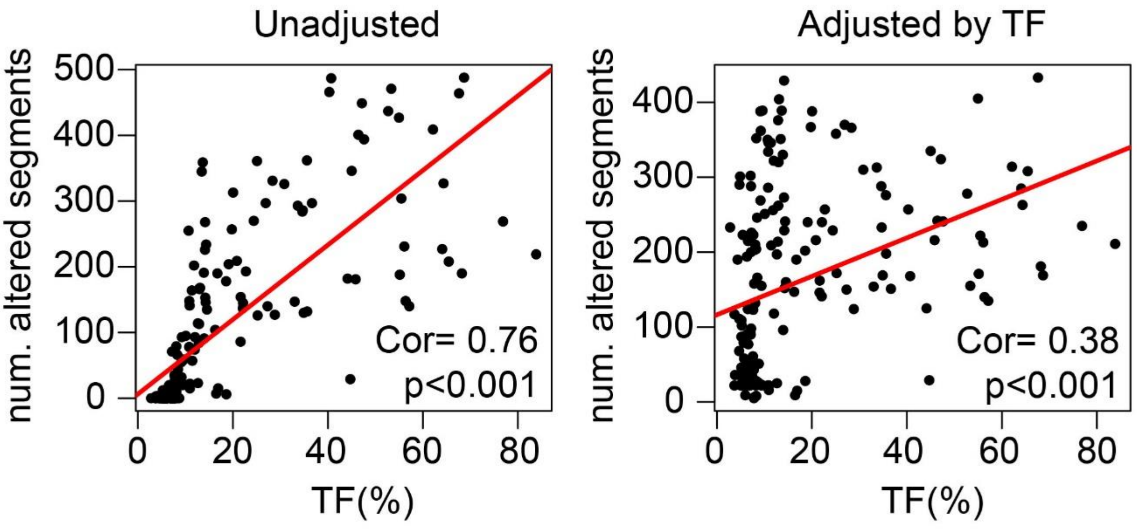

Supplementary Figure 2. Correlation between tumor fraction (TF) and number of altered segments. Relationship between TF and number of altered DNA segments detected across 246 plasma ctDNA samples before (p=2.16e−34) (left) and after (p=1.39e−07) (right) adjusting the DNA copy-number signal by TF and ploidy using the ichorCNA tool. Correlation coefficients (Cor) and p-values were determined by Pearson correlation. Source data are provided as a Source Data file.

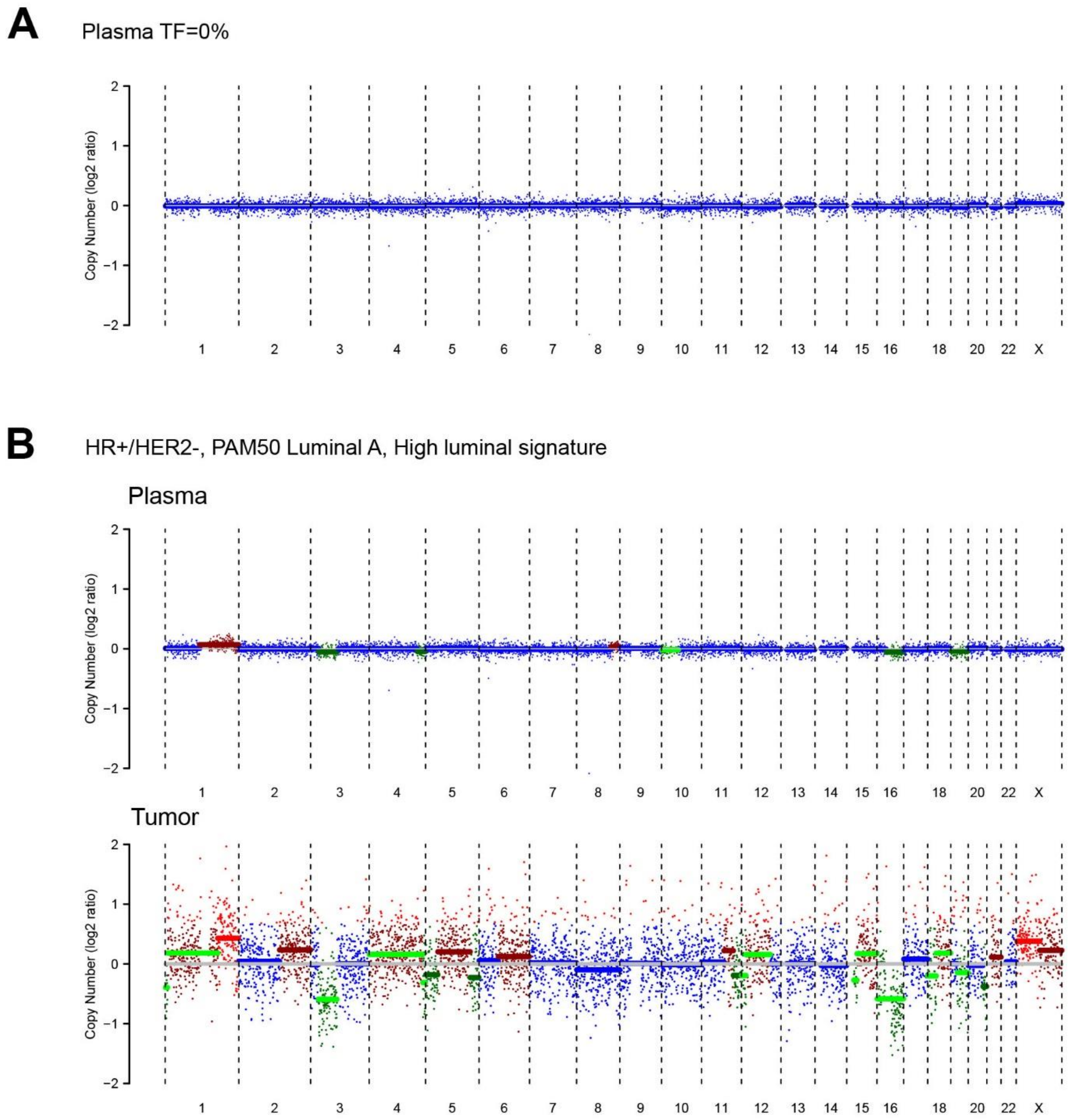

Supplementary Figure 3. Plasma and tissue DNA-based profiles. (A) Example of a copy number alterations (CNA) plot of a plasma sample with TF<3% **(B)** Example of CNA plots of matched timepoint plasma and tissue samples of a HR+/HER2-negative tumour, PAM50 luminal A and high luminal signature score.

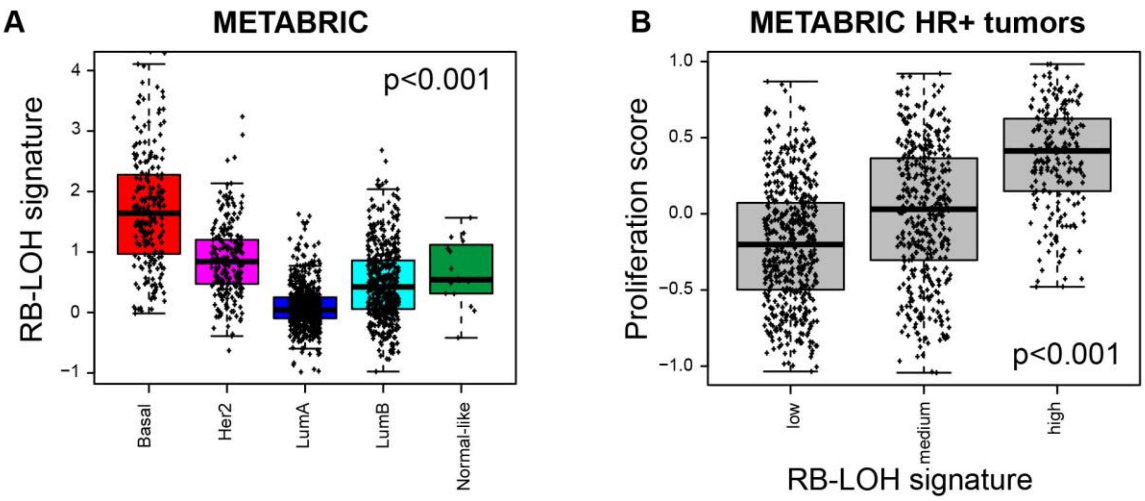

Supplementary Figure 4. Tumor DNA RB-LOH signature relationship with the PAM50 intrinsic subtypes and proliferation score in the METABRIC cohort. (**A**) ANOVA plot of the DNA RB-LOH signature scores across the 4 PAM50 intrinsic subtypes (n=1,517 tumor samples) (p=9.58e-190). (**B**) ANOVA plot of the PAM50 RNA-based proliferation score across HR+/HER2-negative 1,131 tumor samples with low, medium, and high DNA RB-LOH signature scores (defined based on tertiles) (p=4.2e-58). P-values (p) were determined by one-way ANOVA. For the boxplots, center line indicates median; box limits indicate upper and lower quartiles; whiskers indicate 1.5× interquartile range. Source data are provided as a Source Data file.

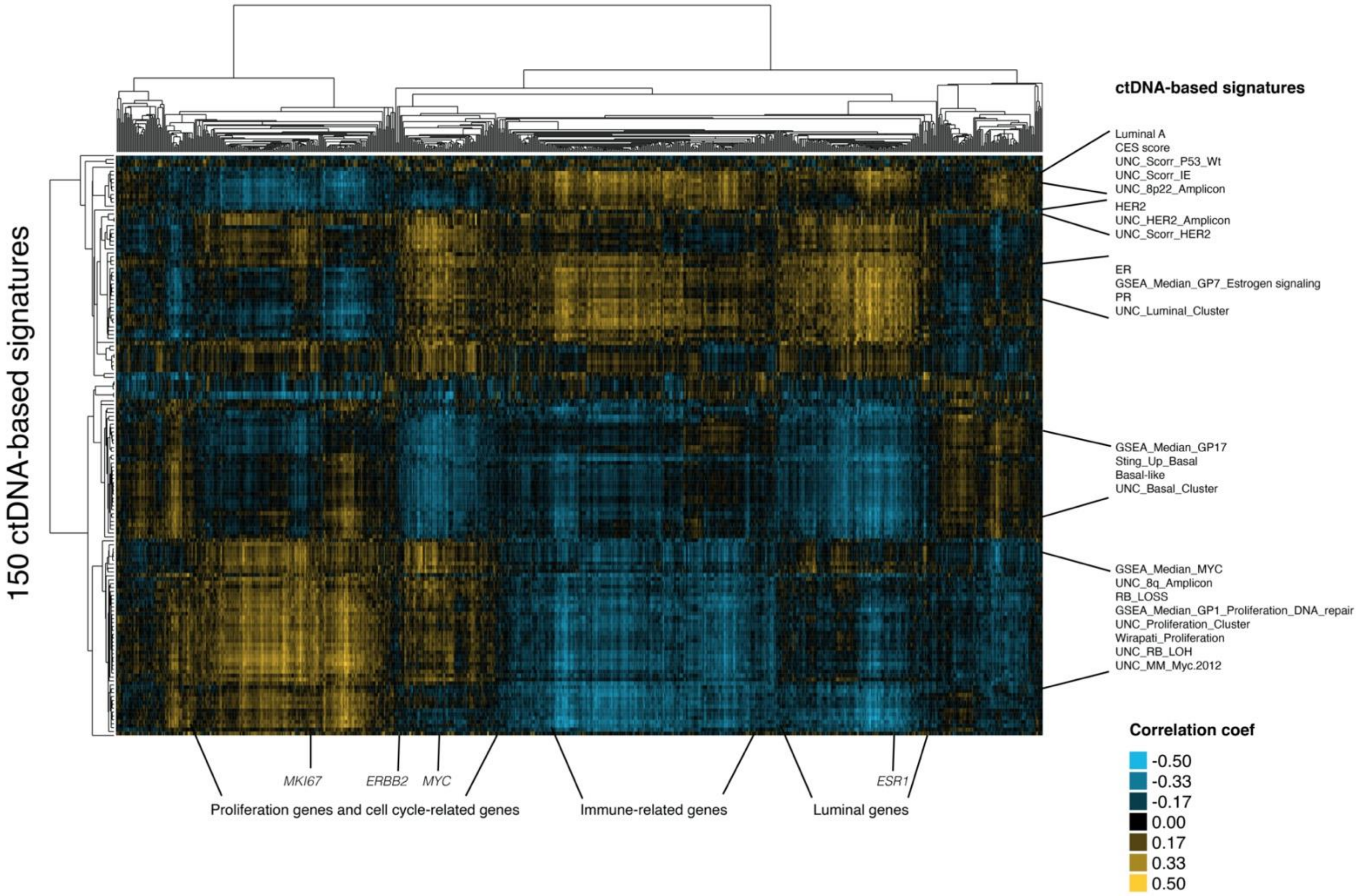

Supplementary Figure 5. Correlation between 150 ctDNA-based signatures and the expression of 771 genes. Unsupervised cluster heatmap analysis of 150 correlation coefficients obtained by comparing the scores of each individual ctDNA-based signature versus the log2 values of the mRNA expression of each of the 771 genes. Source data are provided as a Source Data file.

A

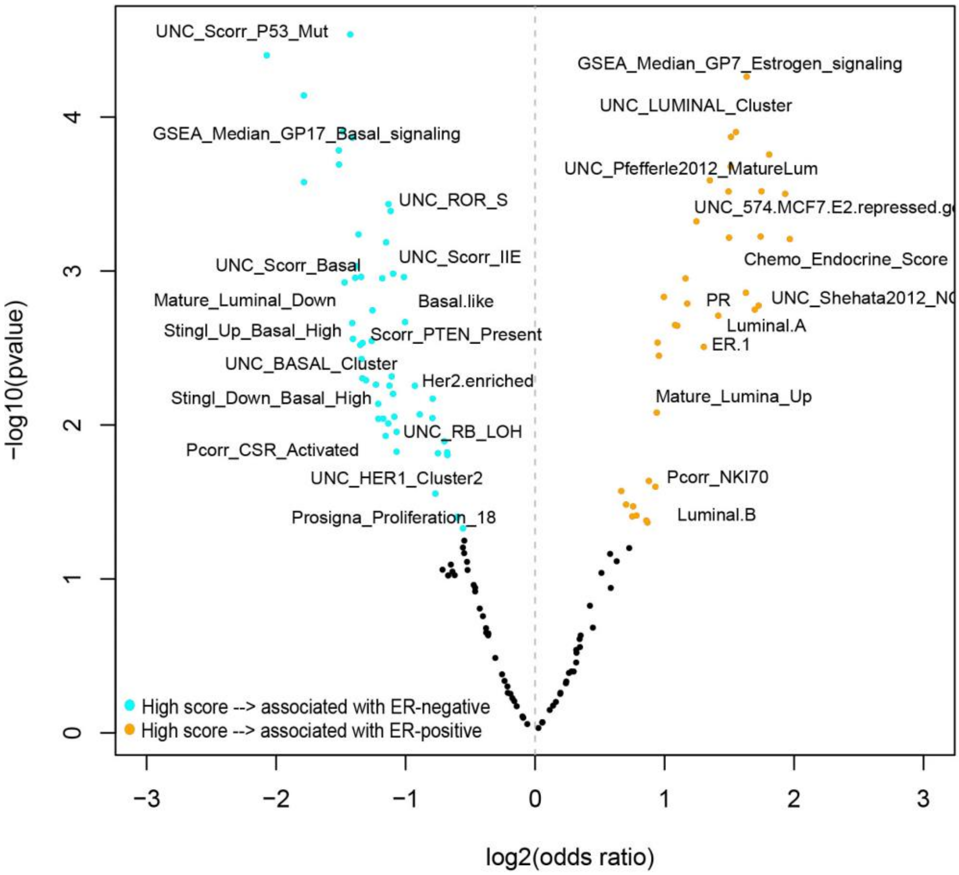

UNC_Scorr_P53_Mut
GSEA_Median_GP17_Basal_signaling
UNC_ROR_S
UNC_Scorr_IIE
UNC_Scorr_Basal
Mature_Luminal_Down
Basal.like
Stingl_Up_Basal_High
Scorr_PTEN_Present
UNC_BASAL_Cluster
Her2.enriched
Stingl_Down_Basal_High
UNC_RB_LOH
Pcorr_CSR_Activated
UNC_HER1_Cluster2
Prosigna_Proliferation_18
GSEA_Median_GP7_Estrogen_signaling
UNC_LUMINAL_Cluster
UNC_Pfefferle2012_MatureLum
UNC_574.MCF7.E2.repressed.g
Chemo_Endocrine_Score
PR
UNC_Shehata2012_N
Luminal.A
ER.1
Mature_Lumina_Up
Pcorr_NKI70
Luminal.B
High score --> associated with ER-negative
High score --> associated with ER-positive
-log10(pvalue)
log2(odds ratio)

B

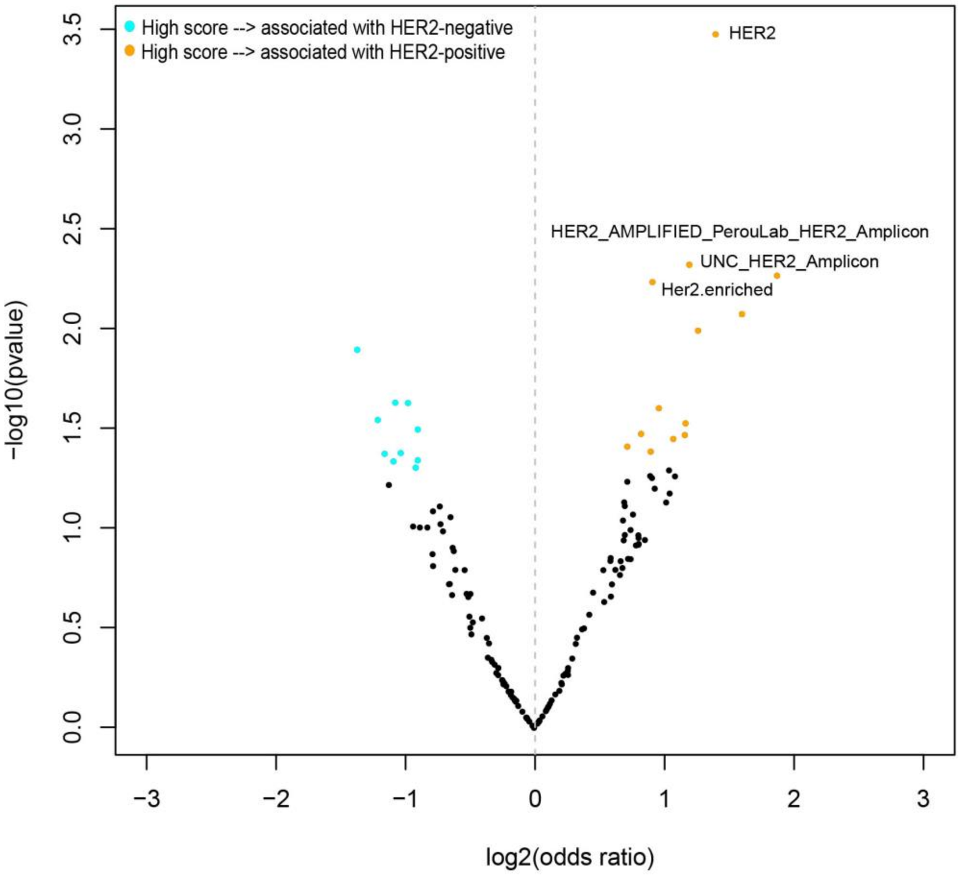

High score --> associated with HER2-negative
High score --> associated with HER2-positive
HER2
HER2_AMPLIFIED_PerouLab_HER2_Amplicon
UNC_HER2_Amplicon
Her2.enriched
-log10(pvalue)
log2(odds ratio)

Supplementary Figure 6. Association of 150 ctDNA-based signatures with ER and HER2 expression in 177 paired ctDNA and tumor samples. Volcano plots illustrating the logistic regression analysis of each ctDNA-based signature score with **(A)** ER (positive or negative) status and **(B)** HER2 (positive or negative) status. Significant scores associated with ER/HER2-positive tumours were orange-coloured while significant scores associated with ER/HER2-negative tumours were cyan-coloured. Odds ratios and p-values were determined by univariate logistic regression analysis. Source data are provided as a Source Data file.

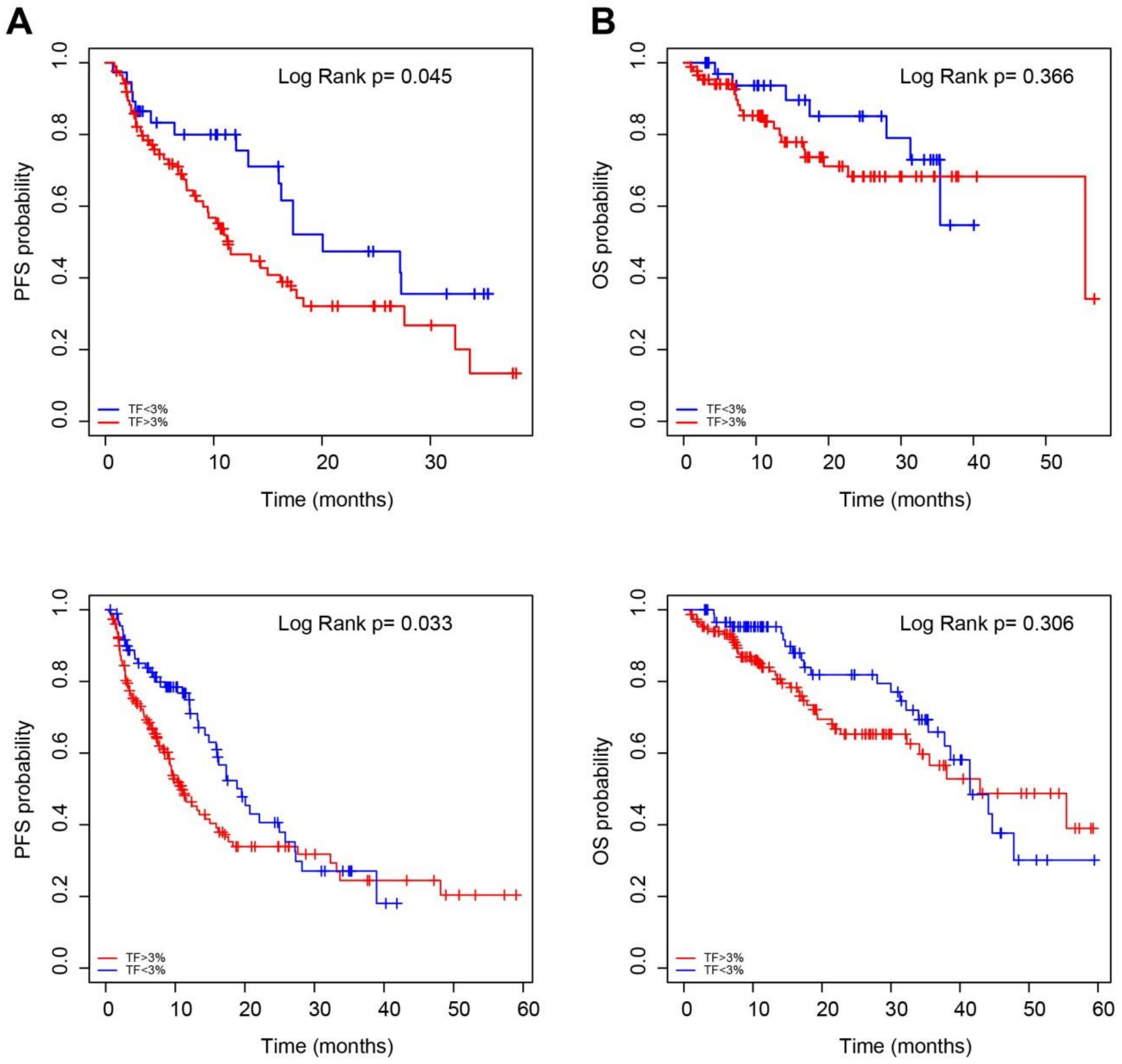

Supplementary Figure 7. Association of TF with PFS and OS in patients with advanced HR+/HER2-negative breast cancer treated with endocrine therapy and CDK4/6 inhibitors. Kaplan-Meier curve of PFS and OS of TF in 124 patients of (**A**) CDK-Validation-1 cohort and **(B)** in 245 patients of CDK-Validation-1 and CDK-Validation-2 cohorts combined. P-values (p) were determined by Log Rank Test. Source data are provided as a Source Data file.

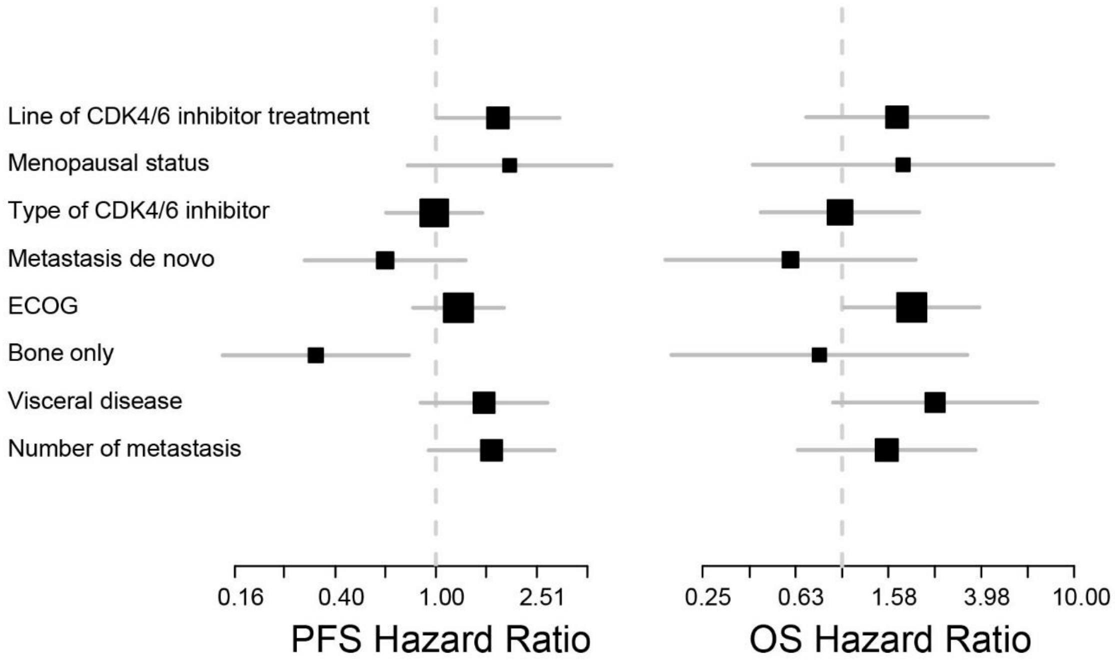

Supplementary Figure 8. Association clinical variables with PFS and OS in 87 patients with advanced HR+/HER2-negative breast cancer treated with endocrine therapy and CDK4/6 inhibitors. Forest plots of hazard ratios (HRs) for PFS (left) and OS (right) of clinical variables in patients of CDK-Validation-1. Data are presented as the hazard ratios with error bars showing 95% confidence intervals. Source data are provided as a Source Data file.

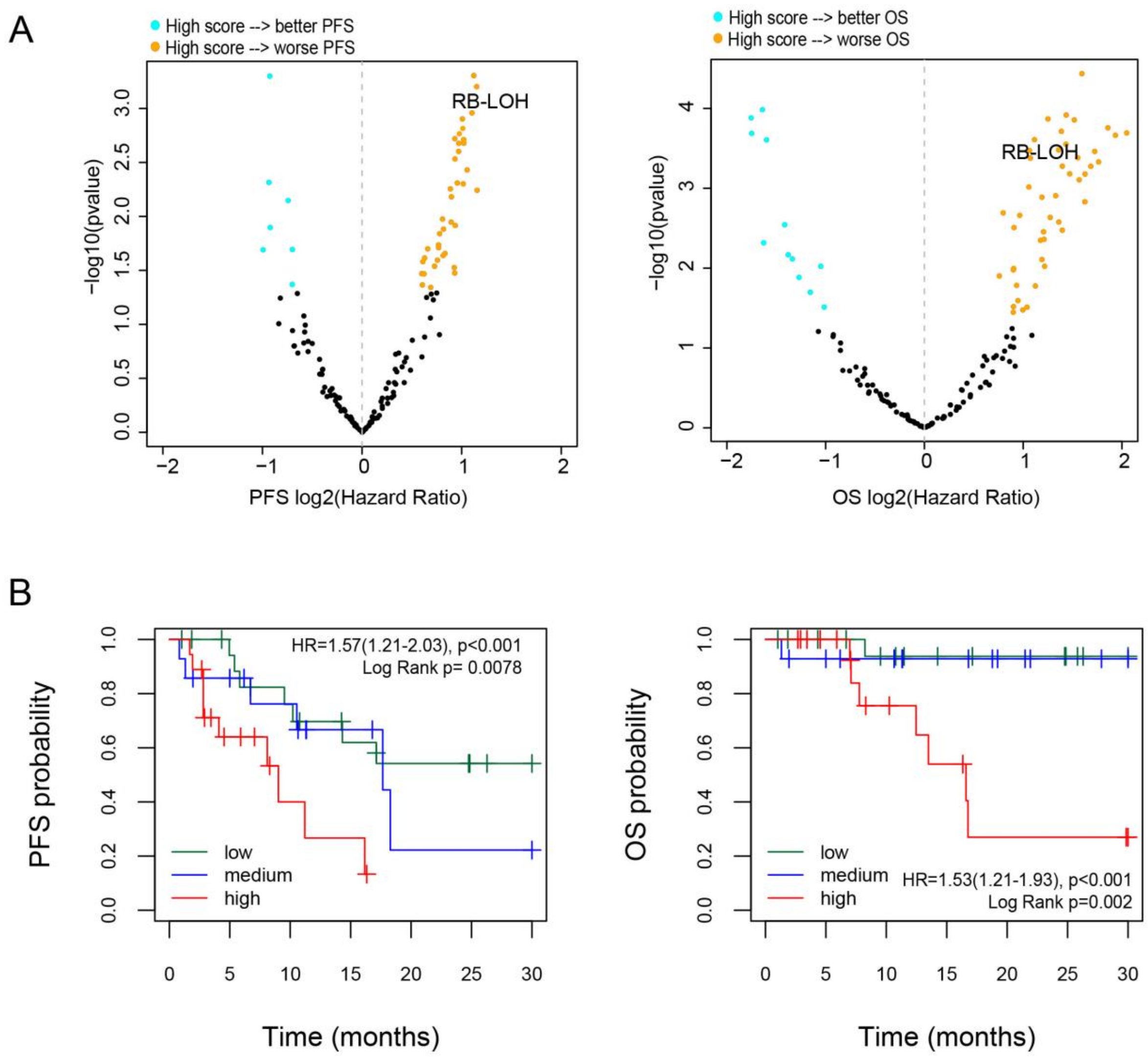

Supplementary Figure 9. Association of tumor ctDNA-based signatures with clinical outcome in 52 patients with advanced HR+/HER2-negative breast cancer treated with endocrine therapy and a CDK4/6 inhibitor in the first-line setting of the CDK-Validation-1 cohort. (A) Volcano plots illustrating the univariate Cox regression analysis of each ctDNA-based signature score (as a continuous variable) with PFS (left) and OS (right). In the volcano plots, significant scores associated with worse survival were orange-colored while significant scores associated with better survival were cyan-colored. Cox models for PFS and OS were used to test the prognostic significance of each variable. Source data are provided in Supplementary Data 7. **(B)** Kaplan-Meier curves of PFS (p=0.00068) (left) and OS (p=0.00038) (right) of the RB-LOH ctDNA-based signature using tertiles as groups. P-values (p) were determined by Log Rank Test and univariate Cox models. Source data are provided as Source Data.

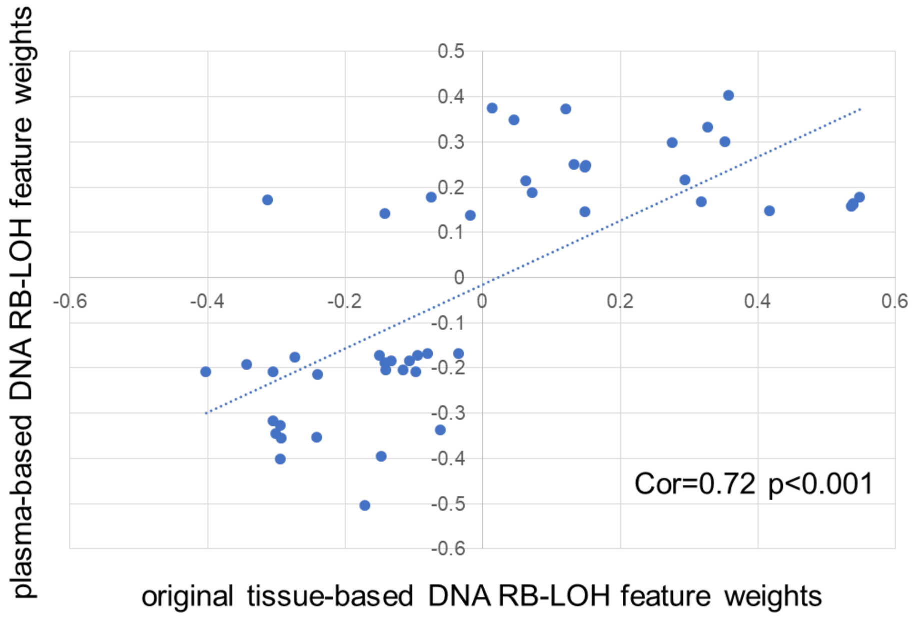

Supplementary Figure 10. Correlation of the 48 main feature weights of the original tissue-based DNA RB-LOH signature and plasma-based DNA RB-LOH signature. Correlation coefficient (Cor) and p-value (p=6.7e-09) were determined by Pearson correlation.

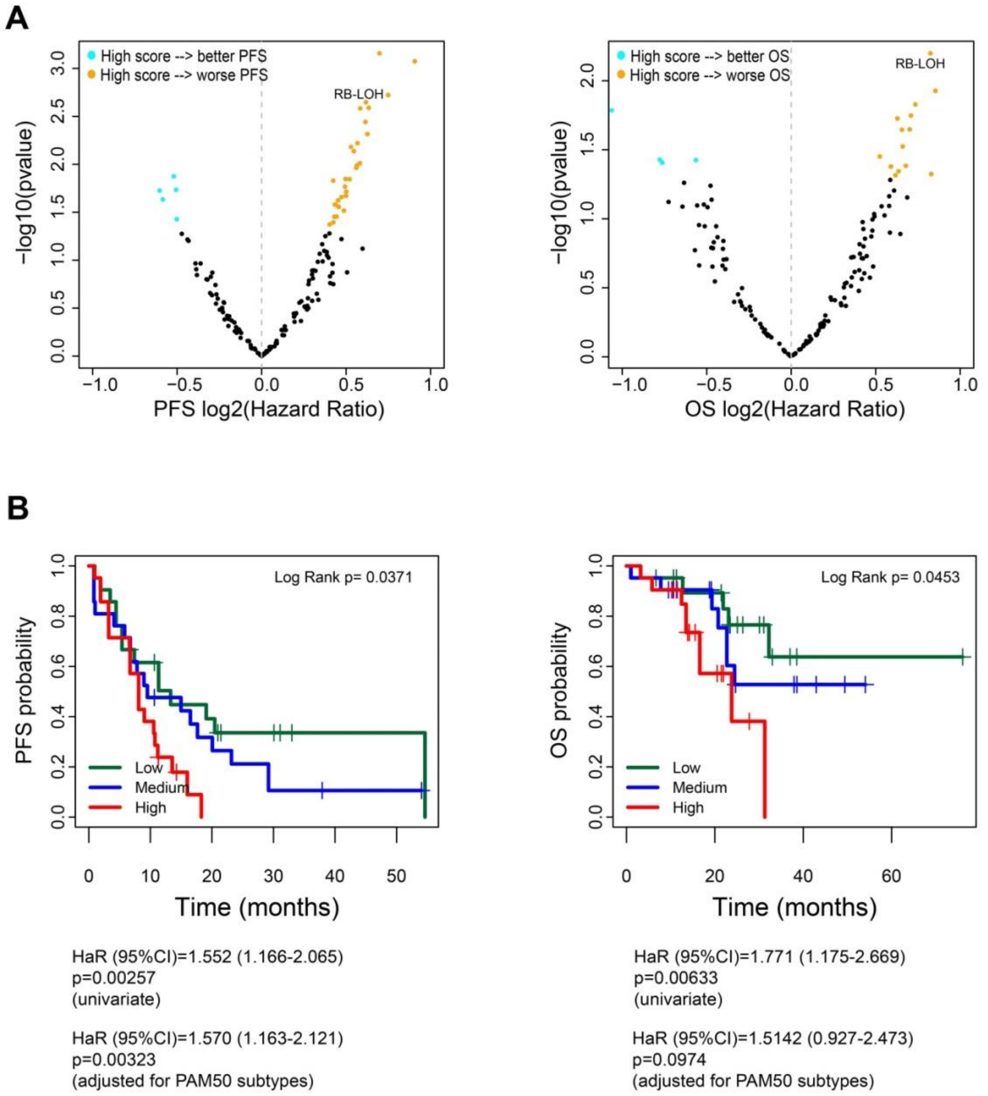

Supplementary Figure 11. Association of tumor DNA-based signatures with clinical outcome in 63 patients with advanced HR+/HER2-negative breast cancer treated with endocrine therapy and CDK4/6 inhibitors. (A) Volcano plots illustrating the univariate Cox regression analysis of each tumor DNA-based signature score (as a continuous variable) with PFS (left) and OS (right). In the volcano plot, significant scores associated with worse survival were orange-coloured while significant scores associated with better survival were cyan-coloured. Cox models for PFS and OS were used to test the prognostic significance of each variable. Source data are provided as Source Data. **(B)** Kaplan-Meier curves of PFS (left) and OS (right) of the RB-LOH tumor DNA-based signature using tertiles. P-values (p) were determined by Log Rank Test and univariate Cox models. Source data are provided as Source Data.

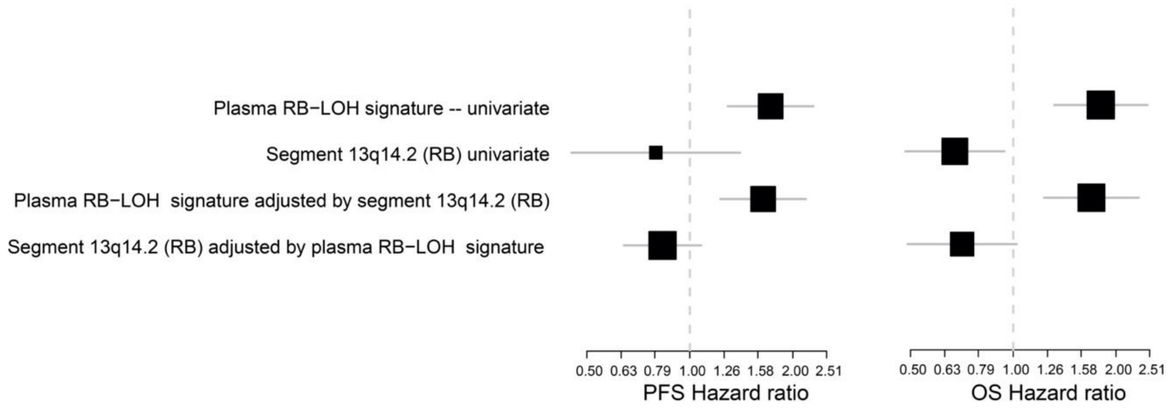

Supplementary Figure 12. Clinical outcome according to ctDNA-based RB-LOH signature and the signal from the ctDNA segment 13q14.2 in advanced HR+/HER2-negative breast cancer treated with endocrine therapy and a CDK4/6 inhibitor. Forest plots of hazard ratios for PFS (left) and OS (right) of the ctDNA-based RB-LOH signature and the signal from the DNA segment 13q14.2 in 87 plasma samples in univariate and bivariate Cox models. Data are presented as the hazard ratios with error bars showing 95% confidence intervals. Source data are provided as Source Data.

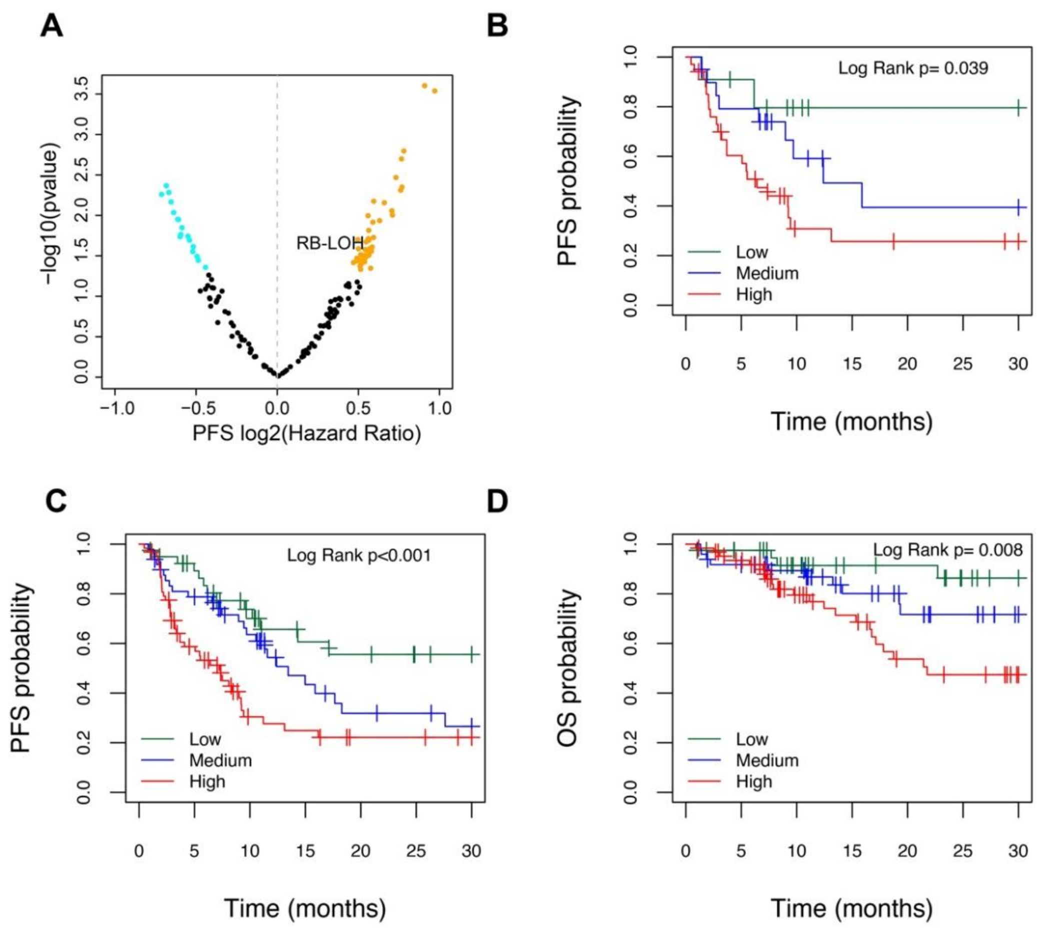

Supplementary Figure 13. Association of ctDNA-based signatures with PFS in patients with advanced HR+/HER2-negative breast cancer treated with endocrine therapy and CDK4/6 inhibitors of a second validation cohort. (A) Volcano plot illustrating the univariate Cox regression analysis of each tumor DNA-based signature score (as a continuous variable) with PFS in 65 patients of the second validation cohort. In the volcano plot, significant scores associated with worse survival were orange-colored while significant scores associated with better survival were cyan-colored. Cox models for PFS and OS were used to test the prognostic significance of each variable. **(B)** Kaplan-Meier curves of PFS of the RB-LOH ctDNA-based signature using the pre-specified RB-LOH signature cutoffs in the second validation cohort of 65 patients. **(C)** Kaplan-Meier curves of PFS of the RB-LOH ctDNA-based signature using the pre-specified RB-LOH signature cutoffs in a combined dataset of 152 patients of both CDK-Validation-1 and CDK-Validation-2 (Log Rank p=0.0006). **(D)** Kaplan-Meier curves of OS of the RB-LOH ctDNA-based signature using the pre-specified RB-LOH signature cutoffs in a combined dataset of 152 patients of both CDK-Validation-1 and CDK-Validation-2. P-values (p) were determined by Log Rank Test. Source data are provided as Source Data.

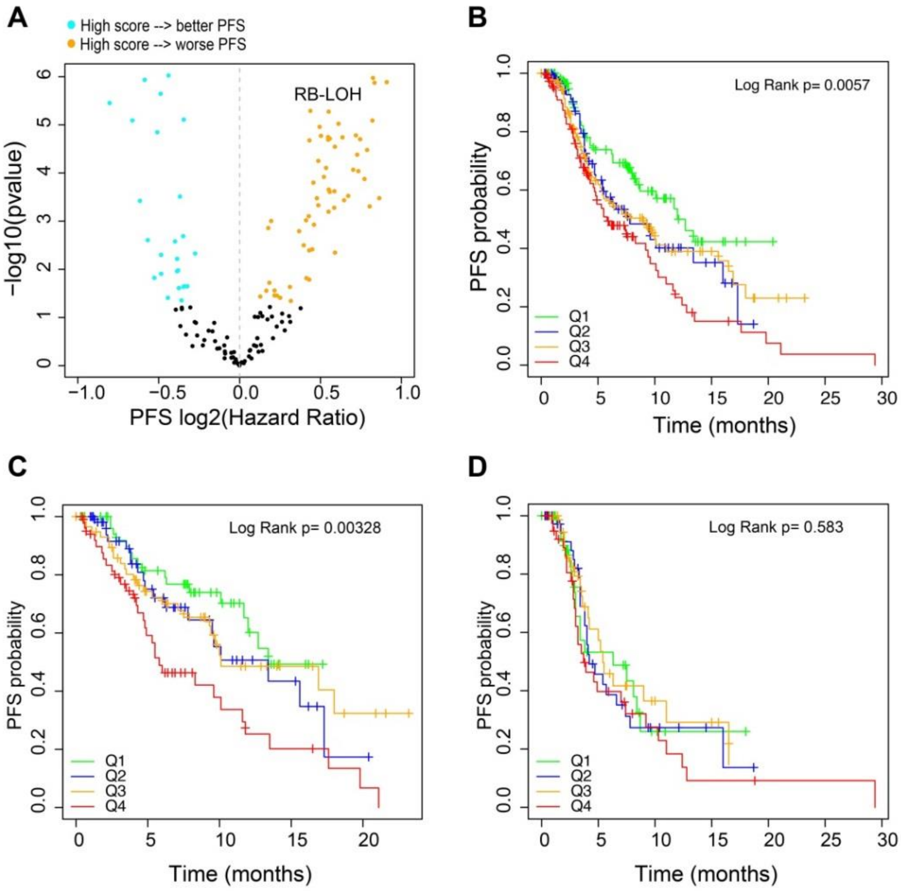

Supplementary Figure 14. Association of tumor DNA-based signatures with PFS in 381 patients with advanced HR+/HER2-negative breast cancer treated with endocrine therapy and CDK4/6 inhibitors of the MSKCC cohort. (A) Volcano plot illustrating the univariate Cox regression analysis of each tumor DNA-based signature score (as a continuous variable) with PFS. In the volcano plot, significant scores associated with worse survival were orange-colored while significant scores associated with better survival were cyan-colored. Cox models for PFS and OS were used to test the prognostic significance of each variable. **(B)** Kaplan-Meier curves of PFS of the RB-LOH tumor DNA-based signature using quartiles (Q1: lowest quartile – Q4: highest quartile) in 381 patients with advanced HR+/HER2-negative breast cancer treated with endocrine therapy and CDK4/6 inhibitors. **(C)** Kaplan-Meier curves of PFS of the RB-LOH tumor DNA-based signature assessed in 223 patients with tumor biopsies taken less than 1 year before the start of treatment with CDK4/6 inhibitors (Q1: lowest quartile – Q4: highest quartile). **(D)** Kaplan-Meier curves of PFS of the RB-LOH tumor DNA-based signature assessed in 158 patients with tumor biopsies taken more than 1 year before the start of treatment with CDK4/6 inhibitors (Q1: lowest quartile – Q4: highest quartile). P-values (p) were determined by Log Rank Test. Source data are provided as Source Data.

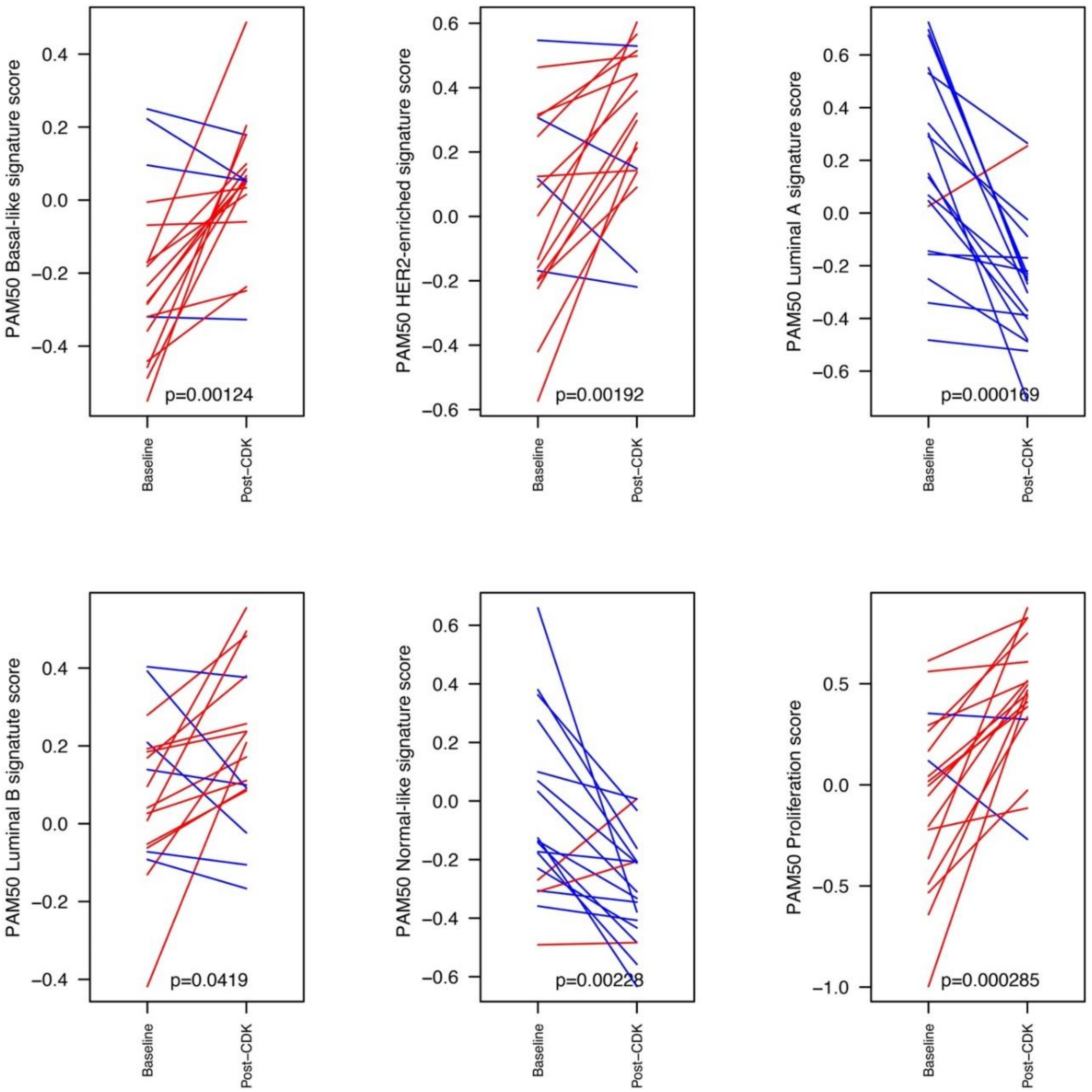

Supplementary Figure 15. PAM50 signatures determined in paired FFPE tumor samples before and after treatment with endocrine therapy and CDK4/6 inhibitors. PAM50 RNA-based signatures at baseline and progressive disease to treatment with endocrine therapy and CDK4/6 inhibitors in 18 paired samples. P-values (p) were determined by a two tailed Student´s t-test. Source data are provided as Source Data.

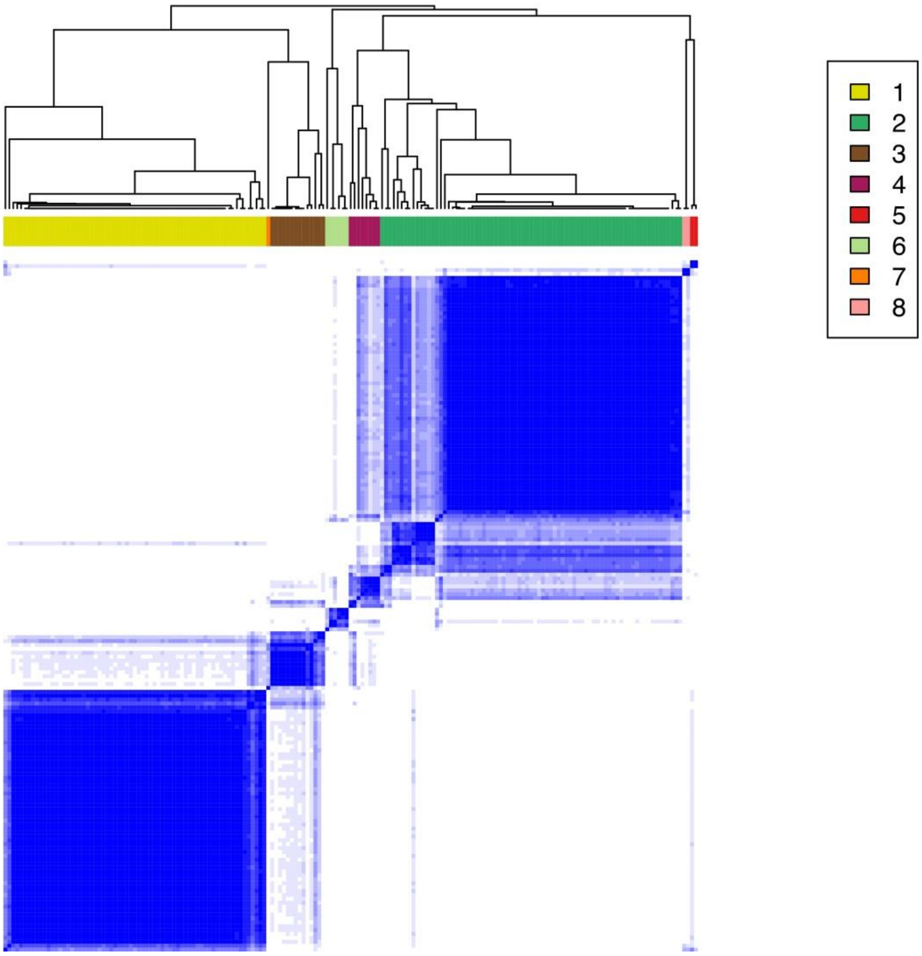

Supplementary Figure 16. Unsupervised classification identified 4 main clusters with a minimum of ≥8 samples in each cluster. Consensus matrix for k = 8 obtained by applying consensus clustering. The colour gradients represent the degree of consensus from 0 to 1, with white corresponding to 0 and blue to 1.

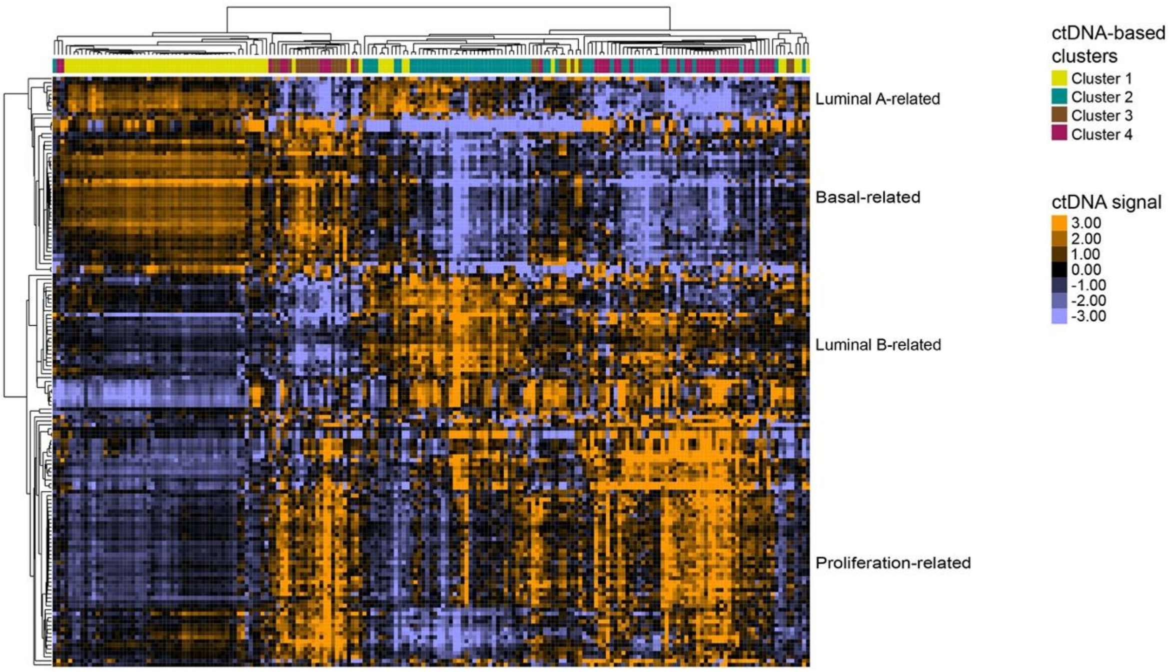

Supplementary Figure 17. ctDNA-based tumor profiles in plasma samples of the Plasma-2 cohort. Unsupervised cluster analysis of 192 plasma samples (columns) of patients with advanced breast cancer and the 150 DNA-based signatures scores (rows). Orange and violet colors represent scores above and below the median value of the signature across the dataset. Below the array tree, the 4 clusters are show. Source data are provided as Source Data.

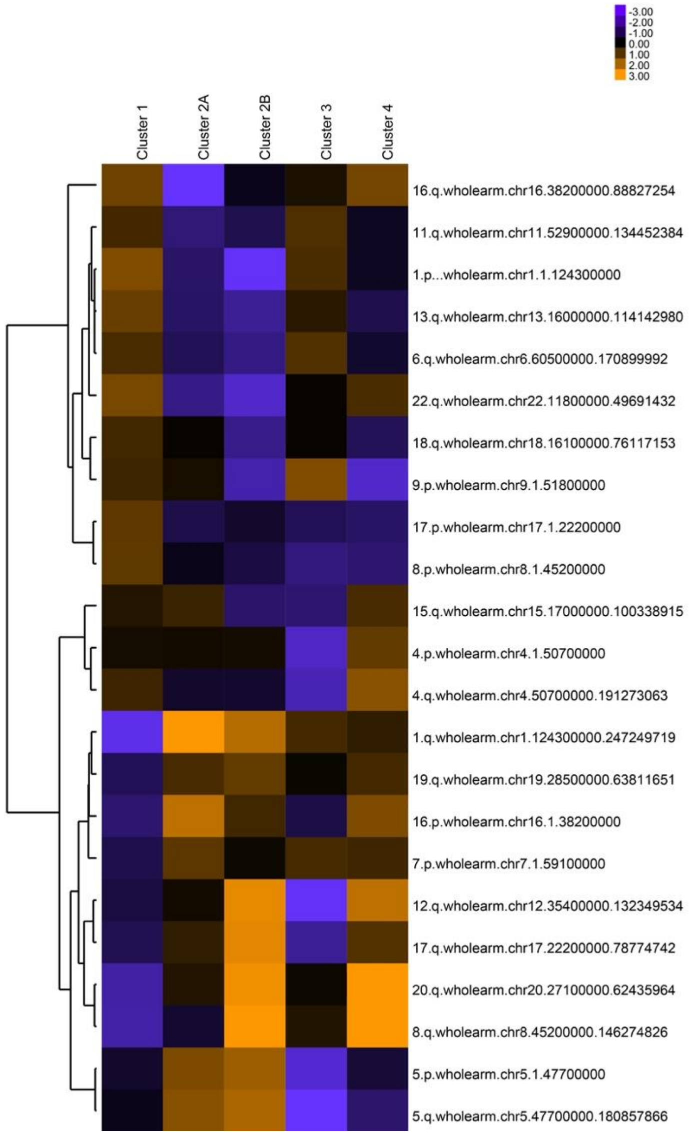

Supplementary Figure 18. Representative heatmap of chromosomic regions associated with each cluster. Differentially represented segments were determined using quantitative SAM analysis (FDR<5%). Source data are provided in Supplementary Data 10.

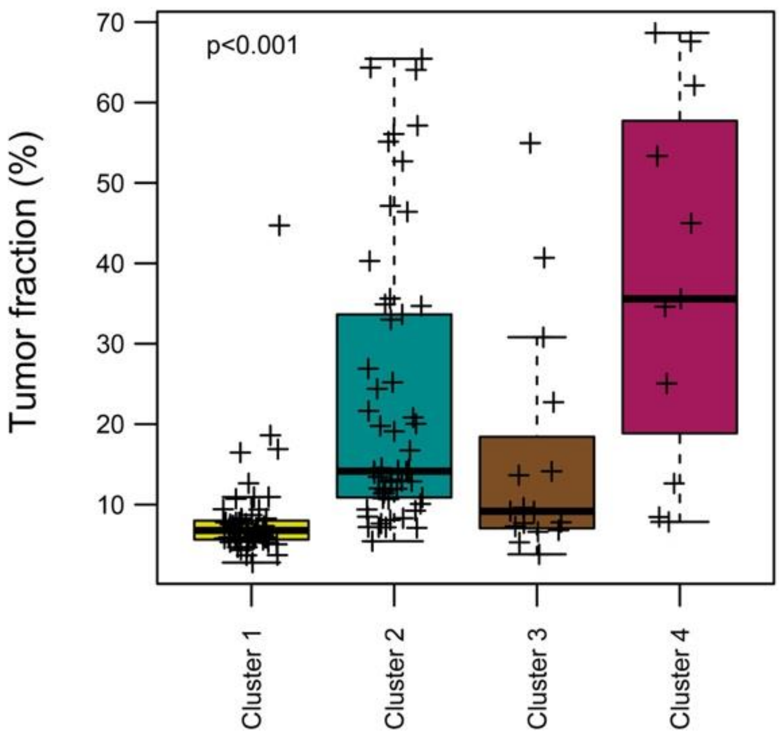

Supplementary Figure 19. TF fraction across clusters. Boxplot representing TF across clusters (p=2.19e-05) in 141 samples with TF>3%. P-values (p) were determined by one-way ANOVA. For the boxplots, center line indicates median; box limits indicate upper and lower quartiles; whiskers indicate 1.5× interquartile range. Source data are provided as a Source Data file.

A

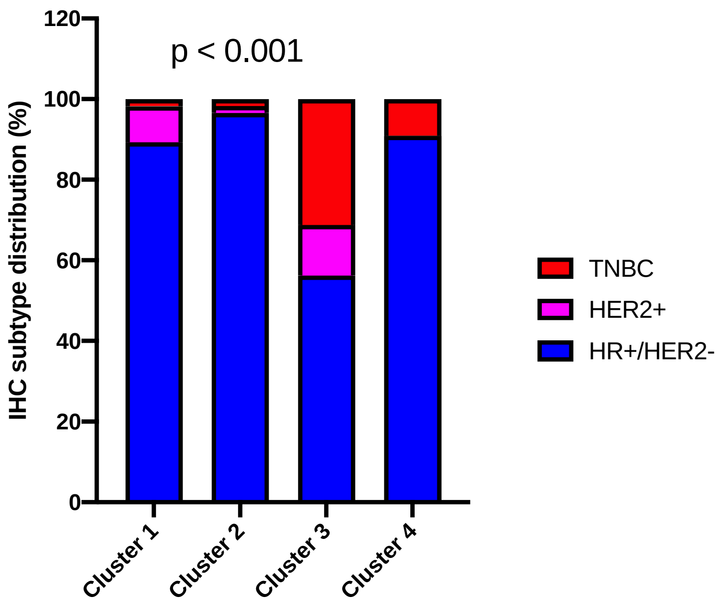

B

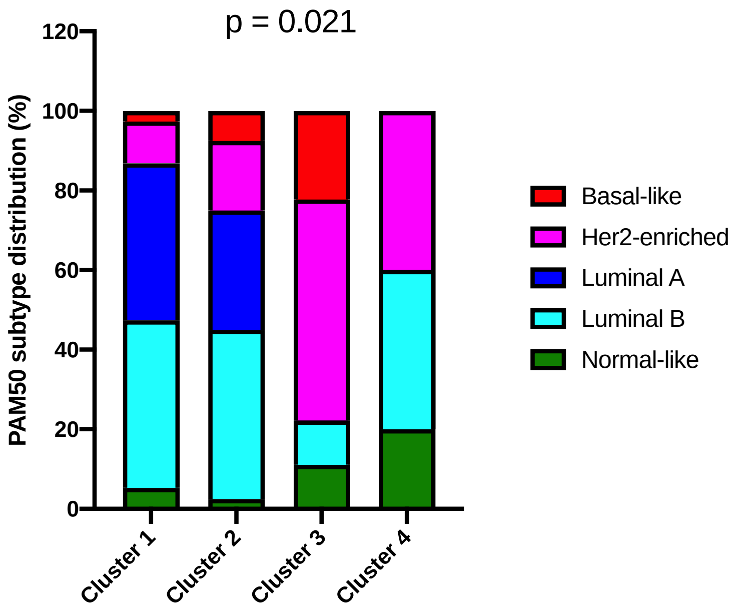

Supplementary Figure 20. Distribution of IHC and PAM50 subtypes in each cluster. Fisher's exact test determined statistical significance of distribution of IHC (p=5.97e-06) (**A**) and PAM50 (p=0.021) (**B**) subtypes in each cluster. Source data are provided as a Source Data file.

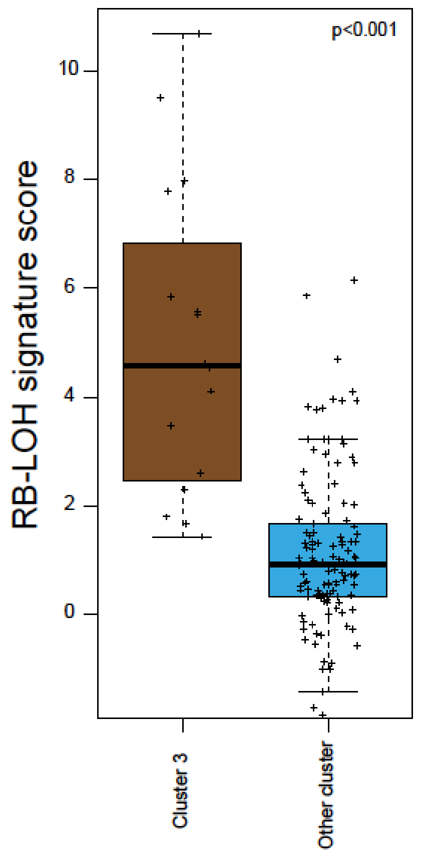

Supplementary Figure 21. RB-LOH signature score in cluster 3. RB-LOH signature score in cluster 3 (n=16) versus the other clusters (n=125) (p=2.05e−14). P-value (p) was determined by two-tailed unpaired t-test. For the boxplots, center line indicates median; box limits indicate upper and lower quartiles; whiskers indicate 1.5× interquartile range. Source data are provided as a Source Data file.

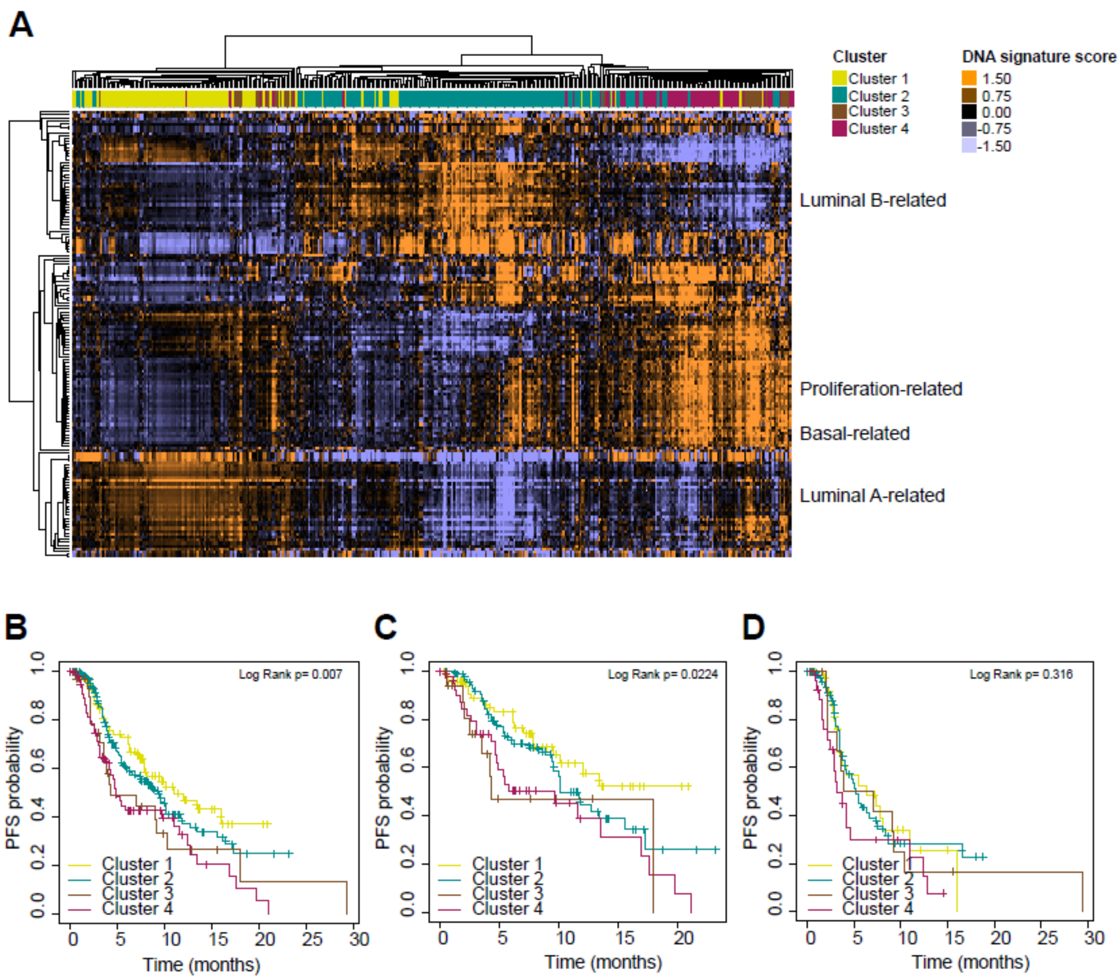

Supplementary Figure 22. DNA-based tumor profiles in tissue samples and association with clinical outcome in the MSKCC publicly available dataset. (A) Unsupervised cluster analysis of 381 tumor samples (columns) of patients with advanced HR+/HER2- breast cancer treated with a CDK4/6 inhibitor and endocrine therapy from the MSKCC dataset and the 150 DNA-based signatures scores (rows). Orange and violet colors represent scores above and below the median value of the signature across the dataset. Below the array tree, the 4 clusters are shown **(B)** Kaplan-Meier curves of PFS (left) and OS (right) of the 4 DNA-based clusters assessed in 381 tumor samples of patients with advanced HR+/HER2- breast cancer treated with a CDK4/6 inhibitor and endocrine therapy from the MSKCC dataset; **(C)** Kaplan-Meier curves of PFS of the 4 DNA-based clusters assessed in 223 patients with tumor biopsies taken less than 1 year before the start of treatment with CDK4/6 inhibitors (Q1: lowest quartile – Q4: highest quartile); **(D)** Kaplan-Meier curves of the 4 DNA-based clusters assessed in 158 patients with tumor biopsies taken more than 1 year before the start of treatment with CDK4/6 inhibitors (Q1: lowest quartile – Q4: highest quartile). P-values (p) were determined by Log Rank Test. Source data are provided as Source Data.

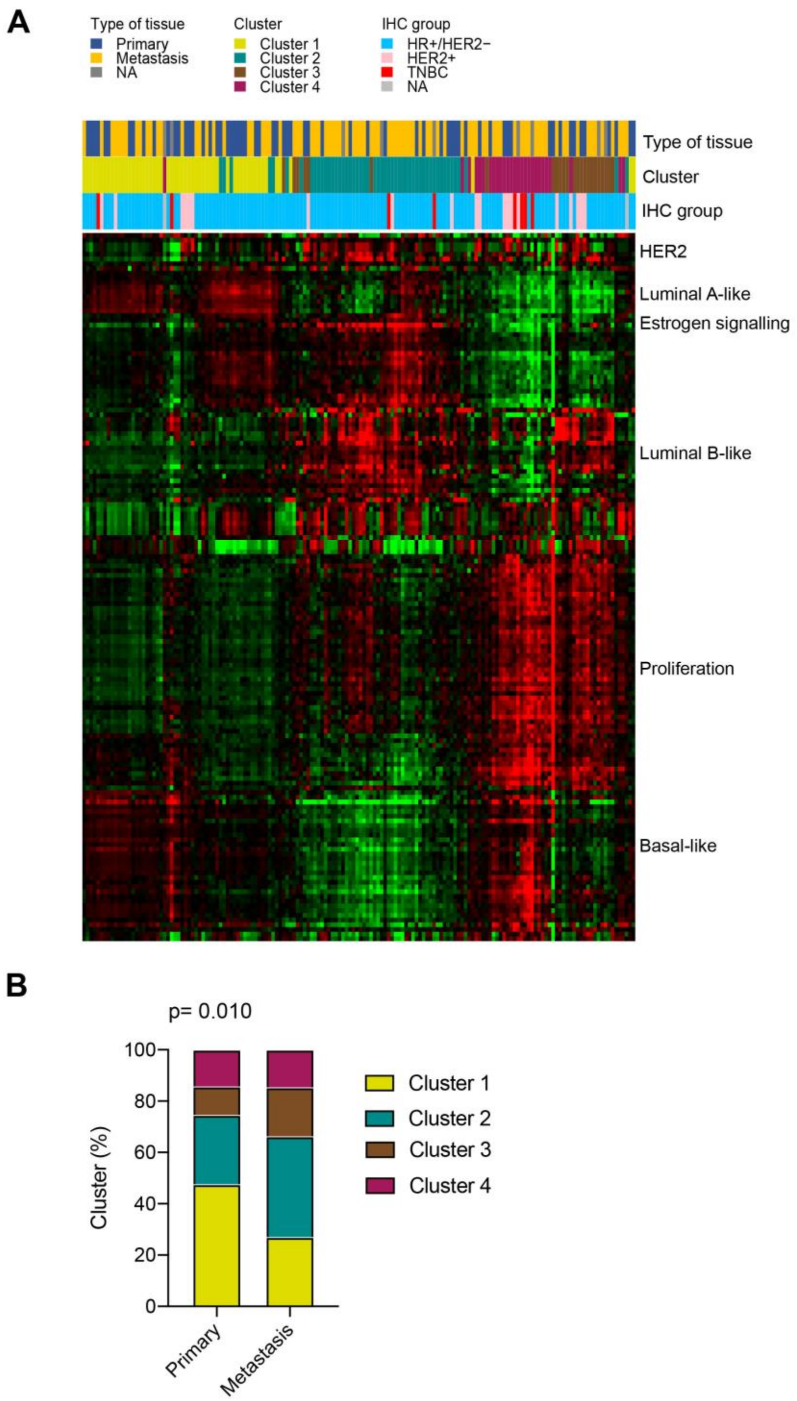

Supplementary Figure 23. Association of DNA-based tumor profiles and clusters in primary and metastatic tissue samples. (A) Unsupervised hierarchical clustering of 150 DNA-based signatures across 158 tumor tissue samples (n=63 primary tumors and 89 metastatic tumors). **(B)** Proportion of cases in each cluster based on tissue-type (primary versus metastatic). Cluster 1: 47.6% in primary vs. 27.0% in metastatic (p-value=0.0103). P-value (p) was determined by Fisher's exact test. Source data are provided as a Source Data file.

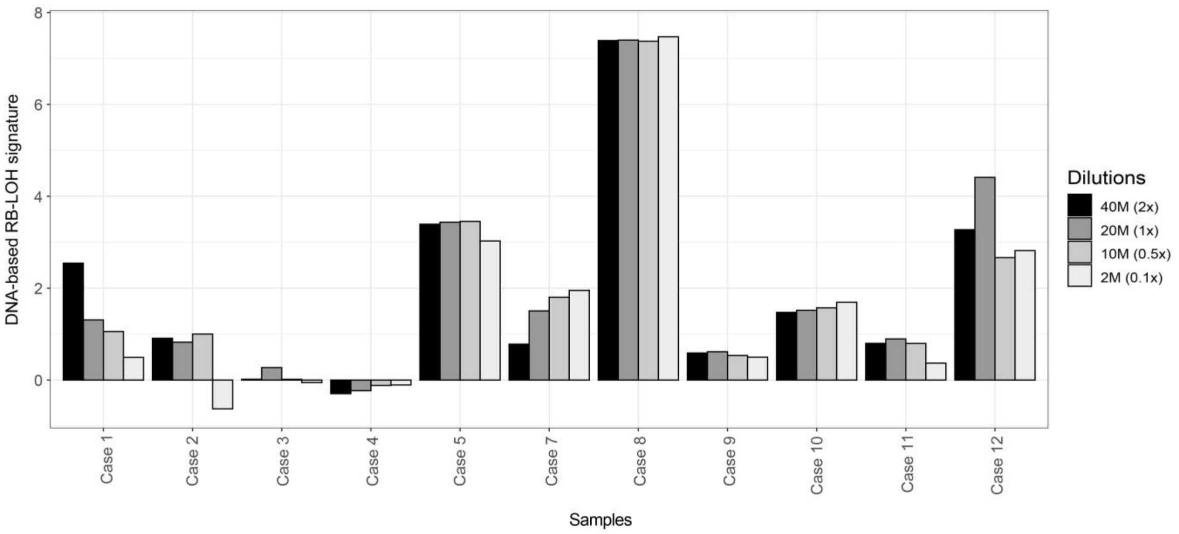

Supplementary Figure 24. RB-LOH signature score using different coverage in 11 samples with TF>3%. Source data are provided as a Source Data file.

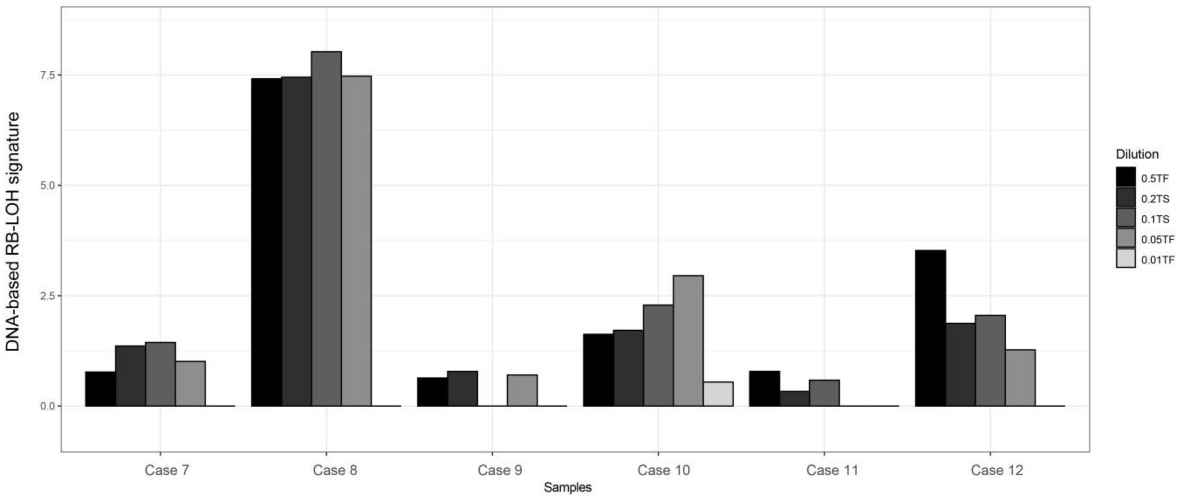

Supplementary Figure 25. RB-LOH signature score in 7 samples that have been in-silico diluted to TFs of 50%, 20%, 10%, 5% and 1% (coverage 0.5X). Source data are provided as a Source Data file.

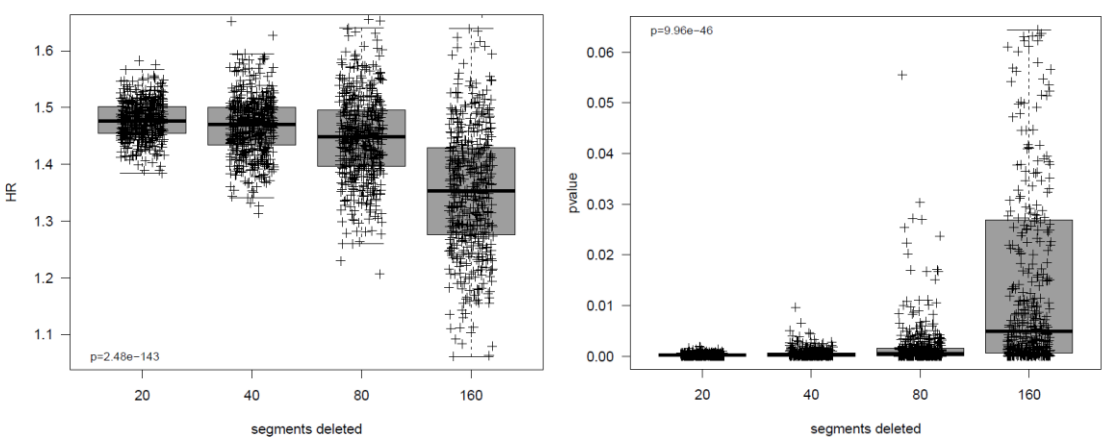

Supplementary Figure 26. PFS hazard ratio and p-value according to the random elimination, for 500 times, of 20, 40, 80 and 160 segments of the 224 segments of the RB-LOH signature. This analysis was carried out in the plasma samples of the 152 patients included in the CDK-Validation-1 and CDK-Validation-2 cohorts. P-values (p) were determined by one-way ANOVA. For the boxplots, center line indicates median; box limits indicate upper and lower quartiles; whiskers indicate 1.5× interquartile range. Source data are provided as a Source Data file.
