# Supplementary material for: Circulating tumor DNA reveals complex biological features with clinical relevance in metastatic breast cancer
Source: Nat Commun. 2023 Mar 1;14:1157. doi: 10.1038/s41467-023-36801-9 (PMC9977734; doi:10.1038/s41467-023-36801-9)
Supplement: Supplementary file 3 — Description of Additional Supplementary Files [file 41467_2023_36801_MOESM3_ESM.docx]

**Description of Additional Supplementary Files**

File Name: Supplementary Data 1

Description: Annotation of gene signatures

File Name: Supplementary Data 2

Description: Annotation of copy number segments

File Name: Supplementary Data 3

Description: Correlation between 150 ctDNA-based signatures and TF in 178 samples with TF>3% without and with TF adjustment

File Name: Supplementary Data 4

Description: Correlation between 150 ctDNA-based signatures and tumor DNA-based signatures

File Name: Supplementary Data 5

Description: Proportion of patients with a correlation coefficient of 514 DNA signals and the 150 CNA-based signatures between paired plasma and tissue

File Name: Supplementary Data 6

Description: Correlation between 150 ctDNA-based signatures scores and PAM50 signature scores

File Name: Supplementary Data 7

Description: Univariate analyses of 150 ctDNA-based signatures for PFS and OS in CDK-Validation-1

File Name: Supplementary Data 8

Description: Univariate analyses of 150 ctDNA-based signatures for PFS and OS in CDK-Validation-1

File Name: Supplementary Data 9

Description: Paired SAM analyses of baseline CDK4/6i treatment and post-CDK4/6i treatment

File Name: Supplementary Data 10

Description: Multiclass SAM analyses of chromosomic regions across clusters

File Name: Supplementary Data 11

Description: Weights of each segment in the 150 ctDNA-based signatures

File Name: Supplementary Data 12

Description: Tumor fraction reported by iChorCNA using shWGS at different coverage depths (2X, 1X, 0.5X, 0.1X)

File Name: Supplementary Data 13

Description: Correlation of bin-to-bin log2 values reported by iChorCNA using shWGS at different coverage depths (2X, 1X, 0.5X, 0.1X)

File Name: Supplementary Data 14

Description: Correlation of 150 CNA signatures run on samples that have been in-silico diluted to different coverage depths

File Name: Supplementary Data 15

Description: Correlation of 150 CNA signatures run on samples that have been in-silico diluted to TFs of 50%, 20%, 10%, 5% and 1% (coverage 0.5X)

File Name: Supplementary Data 16

Description: Statistics of bin log2 values output from iChorCNA of 14 healthy control samples (1Mb bins)

File Name: Supplementary Data 17

Description: Previous treatments of patients of the CDK-Validation 1 cohort were available for 77 patients
