# Supplementary material for: Circulating tumor DNA reveals complex biological features with clinical relevance in metastatic breast cancer
Source: Nat Commun. 2023 Mar 1;14:1157. doi: 10.1038/s41467-023-36801-9 (PMC9977734; doi:10.1038/s41467-023-36801-9)
Supplement: Supplementary file 21 — Reporting Summary [file 41467_2023_36801_MOESM21_ESM.pdf]

nature portfolio

Corresponding author(s): Aleix Prat

Last updated by author(s): Jan 23, 2023

Reporting Summary

Nature Portfolio wishes to improve the reproducibility of the work that we publish. This form provides structure for consistency and transparency in reporting. For further information on Nature Portfolio policies, see our Editorial Policies and the Editorial Policy Checklist.

Statistics

For all statistical analyses, confirm that the following items are present in the figure legend, table legend, main text, or Methods section.

n/a	Confirmed	
☐	☒	The exact sample size (*n*) for each experimental group/condition, given as a discrete number and unit of measurement
☐	☒	A statement on whether measurements were taken from distinct samples or whether the same sample was measured repeatedly
☐	☒	The statistical test(s) used AND whether they are one- or two-sided<br>*Only common tests should be described solely by name; describe more complex techniques in the Methods section.*
☐	☒	A description of all covariates tested
☐	☒	A description of any assumptions or corrections, such as tests of normality and adjustment for multiple comparisons
☐	☒	A full description of the statistical parameters including central tendency (e.g. means) or other basic estimates (e.g. regression coefficient) AND variation (e.g. standard deviation) or associated estimates of uncertainty (e.g. confidence intervals)
☐	☒	For null hypothesis testing, the test statistic (e.g. *F*, *t*, *r*) with confidence intervals, effect sizes, degrees of freedom and *P* value noted<br>*Give P values as exact values whenever suitable.*
☒	☐	For Bayesian analysis, information on the choice of priors and Markov chain Monte Carlo settings
☐	☒	For hierarchical and complex designs, identification of the appropriate level for tests and full reporting of outcomes
☐	☒	Estimates of effect sizes (e.g. Cohen's *d*, Pearson's *r*), indicating how they were calculated

Our web collection on statistics for biologists contains articles on many of the points above.

Software and code

Policy information about availability of computer code

Data collection	No software was used for data collection
Data analysis	All statistical analyses were performed using R software v 4.0.3. ShWGS was analyzed with hmmcopy_utils (https://github.com/shahcompbio/hmmcopy_utils) and ichorCNA v0.2.0 (https://github.com/broadinstitute/ichorCNA). Reads were aligned to the hg19 reference genome with BWA41, applied GATK42 base quality score recalibration, indel realignment and duplicate removal. Variant calling (VarScan2 v2.4.3) required a minimum of 7 reads supporting the variant allele to call a mutation. The sensitivity of the technique is 5% MAF for SNVs and 10% MAF for INDELs. Frequent single nucleotide polymorphisms (SNPs) in the population were removed based on the gnomAD database (allele frequency ≤ 0.0001). Copy number alterations (CNA) were calculated from an in-built genome-wide SNP backbone targeting 20000 SNPs using CNVkit (v0.9.6.dev). Data was manually curated, and classification of identified variants was performed using publicly available databases (COSMIC, cBioPortal, ClinVar, VarSome, OncoKB).

For manuscripts utilizing custom algorithms or software that are central to the research but not yet described in published literature, software must be made available to editors and reviewers. We strongly encourage code deposition in a community repository (e.g. GitHub). See the Nature Portfolio guidelines for submitting code & software for further information.

Data

Policy information about availability of data

All manuscripts must include a data availability statement. This statement should provide the following information, where applicable:

- Accession codes, unique identifiers, or web links for publicly available datasets
- A description of any restrictions on data availability
- For clinical datasets or third party data, please ensure that the statement adheres to our policy

The data collected for the study are not available, as participants of this study did not agree for their data to be shared publicly. However, we encourage investigators interested in data access and collaboration to contact the corresponding author (AP). Access can be obtained under a data transfer agreement and upon Ethics Committee approval. The data generated in this study and presented in the figures are provided in the Supplementary Data/Source Data files. Publicly available DNA, RNA and clinical data from METABRIC and the MSKCC datasets were obtained from cBioportal.

Human research participants

Policy information about studies involving human research participants and Sex and Gender in Research.

Reporting on sex and gender	We have added sex information.
Population characteristics	Reported in Table 2
Recruitment	This was an exploratory analyses and we used all available plasma samples from patients with metastatic breast cancer that had signed an informed consent for the study.
Ethics oversight	The hospital institutional ethics committee approved the study in accordance with the principles of Good Clinical Practice, the Declaration of Helsinki, and other applicable local regulations. Written informed consent was obtained from all patients before enrolment. The medical records were retrospectively reviewed to obtain the necessary clinical data.

Note that full information on the approval of the study protocol must also be provided in the manuscript.

Field-specific reporting

Please select the one below that is the best fit for your research. If you are not sure, read the appropriate sections before making your selection.

☒ Life sciences ☐ Behavioural & social sciences ☐ Ecological, evolutionary & environmental sciences

For a reference copy of the document with all sections, see nature.com/documents/nr-reporting-summary-flat.pdf

Life sciences study design

All studies must disclose on these points even when the disclosure is negative.

Sample size	We did not perform a sample size calculation and we used all the samples available for correlative analyses. This has been added as a limitation in the discussion section: "Second, this was an exploratory study, and no formal sample size calculation was performed. Thus, the lack of a formal design through a pre-planned analysis prohibits inference of negative results".
Data exclusions	Plasma samples with ctDNA tumor fraction below 3% were excluded from some analyses.
Replication	Analyses were repeated at least twice and were performed by two investigators. All attempts at replication were successful.
Randomization	Not relevant
Blinding	Blinding was not relevant to this study, the information of all variables was needed to be available to perform correlative science analyses.

Reporting for specific materials, systems and methods

We require information from authors about some types of materials, experimental systems and methods used in many studies. Here, indicate whether each material, system or method listed is relevant to your study. If you are not sure if a list item applies to your research, read the appropriate section before selecting a response.

Materials & experimental systems

n/a	Involved in the study
☒	☐ Antibodies
☒	☐ Eukaryotic cell lines
☒	☐ Palaeontology and archaeology
☒	☐ Animals and other organisms
☒	☐ Clinical data
☒	☐ Dual use research of concern

Methods

n/a	Involved in the study
☒	☐ ChIP-seq
☒	☐ Flow cytometry
☒	☐ MRI-based neuroimaging
